# Underlying disease is the main risk factor in post‐splenectomy complication risk: Data from a national database

**DOI:** 10.1111/bjh.20114

**Published:** 2025-04-29

**Authors:** Maddalena Casale, Raffaella Colombatti, Manuela Balocco, Paola Corti, Susanna Barella, Giovanna Graziadei, Loredana Farinasso, Tommaso Mina, Simone Cesaro, Tommaso Casini, Fiorina Giona, Saverio Ladogana, Pellegrina Pugliese, Lucia Dora Notarangelo, Antonella Sau, Simone Ferrero, Giovanni Palazzi, Giovanna Russo, Ilaria Lazzareschi, Marilena Serra, Saveria Campisi, Gianluca Boscarol, Elena Facchini, Carlo Baronci, Maria Caterina Putti, Domenico Roberti, Marzia Manilia, Antonio Ivan Lazzarino, Gian Luca Forni, Silverio Perrotta, the Italian Network of Asplenia, Piero Farruggia, Francesca Fioredda, Paola Giordano, Federico Verzegnassi, Maria Luisa Casciana, Assunta Tornesello, Ilaria Capolsini, Paolo Grotto, Paola Maria Grazia Sanna, Luciana Rigoli, Rosanna Di Concilio, Flavia Rivellini, Annamaria Pansanisi, Gloria Colarusso

**Affiliations:** ^1^ Department of the Woman, the Child and General and Specialized Surgery University “Luigi Vanvitelli” Naples Italy; ^2^ Pediatric Hematology‐Oncology Unit, Department of Women’s and Child’s Health Azienda Ospedale‐Università di Padova Padua Italy; ^3^ Microcitemia, delle Anemie Congenite e dei Disordini del Metabolismo del Ferro Ente Ospedaliero Ospedali Galliera Genoa Italy; ^4^ Pediatric Department Scientific Institute for Research and Healthcare (IRCCS) San Gerardo dei Tintori Foundation Monza Italy; ^5^ Microcitemia and Rare Anemia Centre – ASL8 Cagliari Italy; ^6^ SC Medicina ad Indirizzo Metabolico Scientific Institute for Research and Healthcare (IRCCS) Ca’ Granda Ospedale Maggiore Policlinico Milan Italy; ^7^ Paediatric Haematology Unit, Department of Paediatrics University Hospital “Città Della Salute e della Scienza” Torino Italy; ^8^ Hemoglobinopathies Unit, Pediatric Hematology/Oncology Scientific Institute for Research and Healthcare (IRCCS) Policlinico San Matteo Pavia Italy; ^9^ Pediatric Hematology Oncology, Department of Mother and Child Azienda Ospedaliera Universitaria Integrata Verona Verona Italy; ^10^ Meyer Children’s Hospital IRCCS Florence Italy; ^11^ Department of Translational and Precision Medicine Sapienza University of Rome Rome Italy; ^12^ Pediatric Onco‐Hematology Unit “Casa Sollievo della Sofferenza” Hospital, IRCCS San Giovanni Rotondo Italy; ^13^ Immunohematology and Transfusion Medicine Unit Policlinico Umberto 1, Sapienza Università di Roma Rome Italy; ^14^ Direzione Medica di Presidio, Children’s Hospital ASST‐Spedali Civili Brescia Italy; ^15^ Pediatric Onco‐Hematology Unit Spirito Santo Hospital Pescara Italy; ^16^ Department of Molecular Biotechnology and Health Science University di Turin Turin Italy; ^17^ Pediatric Hematology and Oncology Azienda Ospedaliero Universitaria di Modena Modena Italy; ^18^ Pediatric Hematology and Oncology Unit Azienda Policlinico “Rodolico‐San Marco”, University of Catania Catania Italy; ^19^ Department of Woman and Child Health and Public Health Agostino Gemelli University Polyclinic IRCCS Rome Italy; ^20^ Internal Medicine Unit, Thalassemia Centre “Fazzi” Hospital Lecce Italy; ^21^ Thalassemia Centre Umberto I Hospital Siracusa Italy; ^22^ Department of Pediatrics Central Teaching Hospital Bolzano Italy; ^23^ Pediatric Oncology and Hematology Scientific Institute for Research and Healthcare (IRCCS) Azienda Ospedaliero‐Universitaria di Bologna Bologna Italy; ^24^ Department of Onco‐Hematology and Cell and Gene Therapy Scientific Institute for Research and Healthcare (IRCCS), Childrens’ Hospital Bambino Gesù Rome Italy; ^25^ EPISTATA, Agency for Clinical Research and Medical Statistics London UK; ^26^ For Anemia Foundation Genoa Italy

**Keywords:** children, infection, post‐splenectomy complications, splenectomy, thrombosis

## Abstract

Splenectomy is required for many haematological conditions and causes an increased risk of severe infections and vascular events. The association between underlying haematological disease, age at splenectomy and post‐splenectomy complications was explored among 1348 splenectomized patients, followed with a median follow‐up time of 13 years and affected by transfusion‐dependent thalassaemia, non‐transfusion‐dependent thalassaemia (NTDT), sickle cell anaemia (SCA), congenital haemolytic anaemias, autoimmune haematological disorders and trauma. Our main statistical approach was based on interaction analyses within competing‐risk survival models. The baseline risk profile differed across diagnostic categories, with SCA being particularly susceptible to infectious complications and NTDT and SCA to vascular events (*p* < 0.001). The age at splenectomy did not impact on infectious risk but rather older age at splenectomy was associated with increased risk for vascular complications. Furthermore, the risk of developing a post‐splenectomy complication was persistent throughout the observation period and not limited to the first 2–3 years after splenectomy. The probability of a post‐splenectomy complication was highly dependent on the underlying disease and not on the age at splenectomy, so the indications for splenectomy must be based on careful assessment of pros and cons in the individual disease, with no need to delay surgery after a certain age when clinically indicated.

## Graphical abstract

The association between underlying haematological disease, age at splenectomy and post‐splenectomy complications was explored among 1348 splenectomized patients, followed in 53 clinical centres with a median follow‐up time of 13 years and affected by transfusion‐dependent thalassaemia (TDT), non‐transfusion‐dependent thalassaemia (NTDT), sickle cell anaemia (SCA), congenital haemolytic anaemias (CHA), autoimmune haematological diseases (AHD) and trauma. The probability of a post‐splenectomy complication was highly dependent on the underlying disease, with SCA being particularly susceptible to infectious complications and NTDT and SCA to vascular events (*p* < 0.001). The age at splenectomy did not impact infectious risk but rather older age at splenectomy was associated with increased risk for vascular complications. Furthermore, the risk of developing a post‐splenectomy complication was persistent throughout the observation period and not limited to the first 2–3 years after splenectomy.

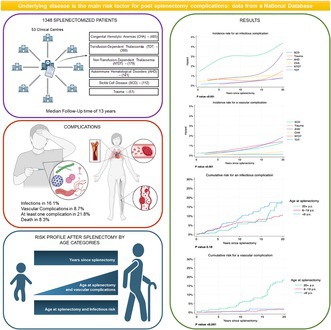

## INTRODUCTION

Surgical removal of the spleen improves or eliminates the symptoms of the underlying condition for many haematological diseases.^1^ However, the resulting asplenia is associated with an increased risk of infectious and vascular complications in the long term.

Splenectomy is indicated in different clinical settings to manage acute or chronic complications of specific haematological diseases, such as hypersplenism and the increased need for blood transfusions in several congenital anaemias.^2^, ^3^, ^4^, ^5^, ^6^ Failure of pharmacological first‐line therapies in haematological autoimmune disorders may also require splenectomy.^7^, ^8^ Splenectomy is urgently required to prevent acute, serious and potentially fatal spleen‐related complications such as acute splenic sequestration in sickle cell anaemia (SCA)^9^, ^10^ and intractable haemorrhage in autoimmune thrombocytopenia.^7^ Subjects with no underlying haematological disorders may require splenectomy for abdominal trauma or for abnormalities of the spleen.

With regard to the complications of asplenia, fulminant and unresponsive‐to‐treatment infections (mainly caused by encapsulated bacteria) facilitated by immune impairment are a widely recognized life‐threatening risk. Historical studies have reported rates of severe post‐splenectomy infections ranging from 1% to 5% of cases, with infection‐related mortality varying between 1.4% and 70% of patients.^11^, ^12^, ^13^

It is expected that the availability of more effective vaccines^14^ and the implementation of infection management recommendations will progressively reduce the risk of infections and infection‐related mortality,^15^ but the impact of these factors is still unknown, due to the lack of contemporary data on clinical outcome rates in splenectomised patients that can guide the clinical decision‐making process.

The risk of post‐splenectomy vascular complications is much less characterized. Arterial thrombosis, deep vein thrombosis, acute portal vein thrombosis, pulmonary embolism and subsequent pulmonary hypertension are more frequent in splenectomised patients.^16^ Variable risk in different haematological diseases is due to the persistence of intravascular haemolysis, hypercoagulability and vascular remodelling, which have greater or lesser impact depending on the underlying disease.^17^

The age at which patients undergo splenectomy may influence their risk of infectious and thrombotic complications, but this is still a matter of debate. It has been reported that the lower the age at splenectomy, the greater the risk of infectious complications.^1^, ^11^, ^12^ In clinical practice, it is generally recommended to postpone splenectomy at least after the age of 6 to reduce the infectious risk.^18^ Recently, no increased risk of infection has been found in children with SCA splenectomised before the age of 3, but no similar data are available in other haematological diseases undergoing splenectomy.^9^, ^19^

In addition, the first 2 years following splenectomy have been reported to have the highest risk of complications^20^; therefore, prophylaxis schemes and clinical or laboratory follow‐ups are generally limited to the first few years following splenectomy. However, these recommendations are derived from historical observational studies preceding the most recent prophylactic guidelines, are limited to specific disease groups, consider heterogeneous end‐points and, therefore, may be obsolete and impractical.^11^, ^12^, ^13^

Thus, the specific long‐term risk of post‐splenectomy complications in different diseases and at different ages has not been defined yet, as studies published so far are either limited to the observation of small groups of patients or come from the analysis of hospital discharge registries lacking detailed medical information or have a short follow‐up time.^21^, ^22^ Therefore, the specific post‐splenectomy risk for individual diseases and the long‐term effects of splenectomy versus other therapies (e.g. drug therapies) are still unclear. Thus, at the present time, the decision to perform splenectomy in different haematological disorders is based on generic and anecdotal data on the risk of post‐splenectomy complications.

For all these reasons, the need for national registries of patients undergoing splenectomy that collect detailed and high‐quality clinical data has recently been emphasized, in order to provide solid indications on post‐splenectomy infectious and vascular risk and thus to guide clinical decision‐making.^23^, ^24^

In this long‐term follow‐up study of splenectomised haematological patients from a national database, we aimed to assess the risk of infectious and vascular complications after splenectomy as a function of the underlying disease and of the patient's age at the time of splenectomy, in order to provide reference data on post‐splenectomy risk in major haematological diseases.

## METHODS

### Setting

For this longitudinal observational study, we used data from the Italian Network of Asplenia (INA) database, which was established in Italy in 2014, coordinated by the Centre for Pediatric Hematology and Oncology of the University of Campania ‘Luigi Vanvitelli’ of Naples and sponsored by the Italian Society of Thalassaemia and Haemoglobinopathies (SITE) and the Italian Association of Paediatric Haematology and Oncology (AIEOP). There are currently 108 INA member doctors today, from 53 Italian hospitals located in 42 cities, covering 16 out of the 20 regional health services regions of Italy. The members of the INA research network are haematologists, oncologists, paediatricians, transfusionists or internal medicine physicians, who oversee the collection of patient‐level data for the patients they personally take care of in their medical centres. Demographic and clinical variables were collected by trained research staff using a standardized electronic entry form.

The research protocol was approved by the local ethical committees and by the institutional review boards of all participating centres and was implemented in accordance with the Declaration of Helsinki and the International Council for Harmonisation of Technical Requirements for Registration of Pharmaceuticals for Human Use (ICH) of European Medicines Agency (EMA) guidelines for good clinical practice. All patients received a verbal and written explanation of the aims and procedure of the study, and written informed consent was obtained.

All consecutive adult and paediatric asplenic patients seen at the participating sites are included in the INA database since 2014. Patient inclusion was not dependent on whether asplenic patients were alive at the time of database formation. For this study, data retrieval for all patients was in July 2023.

### Outcome definitions

The definition of a post‐splenectomy infectious complication included sepsis, pneumonia, meningitis or any other infection that required hospitalization or the use of parenteral antibiotics, or that was the primary cause of the patient's death.

A vascular complication was defined as a complete or partial occlusion of an arterial or venous vessel, diagnosed by appropriate imaging, leading or not leading to an acute event or the patient's death. We excluded heart failure from the definition of a vascular complication because in the context of haematological diseases, heart failure is attributable to the iron overload due to chronic blood transfusions.

### Statistical analysis

We used Stata v.18 to carry out all data analyses. Data were cleaned before the analysis: we checked all variables for missing, illogical or implausible values. This also included cross‐checks with related variables (e.g. chronologic orders). Continuous variables were checked for abnormal distributions and outliers.

Two main outcome measures were identified: the incidence of a severe infectious complication (fatal or non‐fatal) and the incidence of a severe vascular complication (fatal or non‐fatal). These outcomes were considered independent from each other, meaning that the occurrence of one did not preclude the occurrence of the other, unless one was fatal.

We used competing‐risk survival analysis to fit two separate models: one for infections and one for vascular complications. Survival time was measured as the number of days passed since splenectomy to the first of either the outcome, censoring, or death for a non‐disease‐specific cause (competing risk). We tested the proportional subhazards assumption by including time interactions on the covariates and by drawing log–log hazard function plots.

For same analyses, the continuous variable age at splenectomy was cut into three groups, that is, 0–5, 6–19 and 20+ years old, which roughly correspond to its tertiles.

We calculated the predicted probability of experiencing an outcome within 10 years following splenectomy using the coefficients from the survival analysis.

## RESULTS

The INA database comprised 1775 asplenic patients, 300 of whom had a form of asplenia that was not due to splenectomy. Our analysis was therefore restricted to the remaining 1475 patients who underwent a surgical splenectomy and formed our cohort. We applied the following exclusion criteria: historic records showing that the outcome(s) occurred before splenectomy (*n* = 17), unknown reason for splenectomy (*n* = 1), underlying diagnostic category being too small to derive reliable estimates (immunodeficiencies, *n* = 10), underlying diagnostic category being outside the scope of the study (cancer, *n* = 60; other non‐haematological conditions, *n* = 31) and unknown censoring date (*n* = 6). The final analytic sample therefore included 1348 patients, accounting for about 23 000 person‐years. During the median 13 years of follow‐up time (range: 1 month–57 years), 217 patients (16.1%) experienced their first post‐splenectomy infection, 118 (8.7%) their first vascular complication, 294 (21.8%) had at least one of them, 41 (3.0%) had both outcomes and 112 (8.3%) died.

Table 1 shows the demographic and clinical characteristics of our cohort by diagnostic category. Sepsis was reported in 14.3% (31/217) cases, meningitis in 0.9% (2/217) and pneumonia in 40.6% (88/217). Other post‐splenectomy infections were reported in 44.2% (96/217) subjects and represented all acute febrile events that required hospitalization and parenteral antibiotic administration and/or led to patient death and occurred at rates ranging from 26.5% to 50% in the different disease groups. The 96 other post‐splenectomy infections were classified as follows: 35 fever of unknown origin (38%), 22 abscesses (23%), 15 renal infections (16%), 11 haemorrhagic or complicated febrile gastrointestinal infections (11%), seven febrile myopericarditis (7%), four osteomyelitis (3%) and two malaria infections (2%).

**TABLE 1:** Characteristics of the study sample by diagnostic group.

Parameter	TDT	NTDT	SCA	CHA	AHD	Trauma	Total	*p* Value
Number (%)	380 (28.2%)	179 (13.3%)	112 (8.3%)	485 (36.0%)	141 (10.5%)	51 (3.8%)	1348 (100%)	
Gender: Male	52.4% (199/380)	52.5% (94/179)	54.1% (60/111)	54.2% (263/485)	50.4% (71/141)	70.0% (35/50)	53.6% (722/1346)	0.27
Median age at splenectomy (IQR)	10.0 (6.4–18.7)	19.4 (10.3–31.2)	9.2 (6.1–13.6)	8.8 (6.4–13.7)	15.2 (8.5–21.2)	11.3 (6.9–15.0)	10.5 (6.7–18.3)	<0.001
Median follow‐up in years (IQR)	23.9 (13.3–39.2)	26.5 (16.4–36.6)	9.4 (3.8–17.9)	9.5 (4.2–16.5)	6.7 (2.3–11.7)	2.9 (1.2–4.9)	12.9 (5.7–26.4)	<0.001
Antibiotic prophylaxis	42.4% (161/380)	35.8% (64/179)	60.7% (68/112)	67.2% (326/485)	54.6% (77/141)	56.9% (29/51)	53.8% (725/1348)	<0.001
Vaccination	98.4% (374/380)	100.0% (179/179)	98.2% (110/112)	98.1% (476/485)	97.2% (137/141)	100.0% (51/51)	98.4% (1327/1348)	0.36
Heparin	9.7% (37/380)	11.2% (20/179)	25.9% (29/112)	18.4% (89/485)	13.5% (19/141)	17.6% (9/51)	15.1% (203/1348)	<0.001
Anti‐platelet prophylaxis	35.8% (136/380)	57.0% (102/179)	41.1% (46/112)	36.9% (179/485)	8.5% (12/141)	11.8% (6/51)	35.7% (481/1348)	<0.001
Anti‐coagulant prophylaxis	5.8% (22/380)	17.3% (31/179)	7.1% (8/112)	2.1% (10/485)	0.0% (0/141)	0.0% (0/51)	5.3% (71/1348)	<0.001
Infectious complications	15.5% (59/380)	23.5% (42/179)	43.8% (49/112)	10.5% (51/485)	8.5% (12/141)	7.8% (4/51)	16.1% (217/1348)	<0.001
Vascular complications	6.3% (24/380)	34.1% (61/179)	11.6% (13/112)	2.9% (14/485)	3.5% (5/141)	2.0% (1/51)	8.8% (118/1348)	<0.001
Death (any cause)	18.9% (72/380)	16.2% (29/179)	4.5% (5/112)	0.4% (2/485)	2.8% (4/141)	0.0% (0/51)	8.3% (112/1348)	<0.001
Death for infection	13.9% (10/72)	10.3% (3/29)	0.0% (0/5)	0.0% (0/2)	100.0% (4/4)		15.2% (17/112)	<0.001
Death for vascular event	4.2% (3/72)	10.3% (3/29)	20.0% (1/5)	0.0% (0/2)	0.0% (0/4)		6.2% (7/112)	0.50

Abbreviations: AHD, autoimmune haematological disease; CHA, congenital haemolytic anaemia; IQR, interquartile range; NTDT, non‐transfusion‐dependent thalassaemia; SCA, sickle cell anaemia; TDT, transfusion‐dependent thalassaemia.

Vaccine coverage was very high and similar in different underlying disorders, showing high adherence to general recommendations on active immunization in splenectomized patients. Antibiotic, heparin and anti‐platelet prophylaxis were differently prescribed among different disorders, reflecting the controversial or elusive indications of these preventative measures in post‐splenectomy.^18^

**FIGURE 1:**
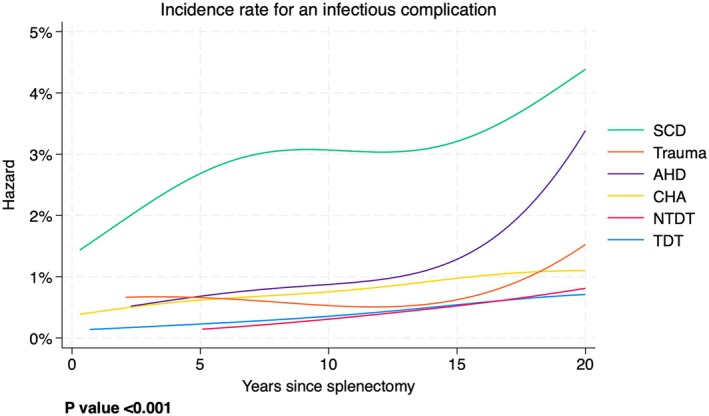
Incidence rate for an infectious complication year by year for each diagnostic group. AHD, autoimmune haematological disease; CHA, congenital haemolytic anaemia; NTDT, non‐transfusion‐dependent thalassaemia; SCA, sickle cell anaemia; TDT, transfusion‐dependent thalassaemia.

Figure 1 shows the risk of an infectious complication year by year for each diagnostic group separately. For example, an SCA patient (green line) who has been free from infectious complications for 10 years after splenectomy had a risk of about 3% to experience an infectious complication before the 11th year. The most important aspects of this graph are the following: (1) the risk never decreases with time, for any disease; (2) SCA patients always have a higher risk than patients with other diseases and (3) the other diseases have a very similar trend up to 10–15 years after splenectomy. The *p* value for a difference between the curves was <0.001, indicating that there is very strong evidence that the risk patterns are different between the disease groups.

**FIGURE 2:**
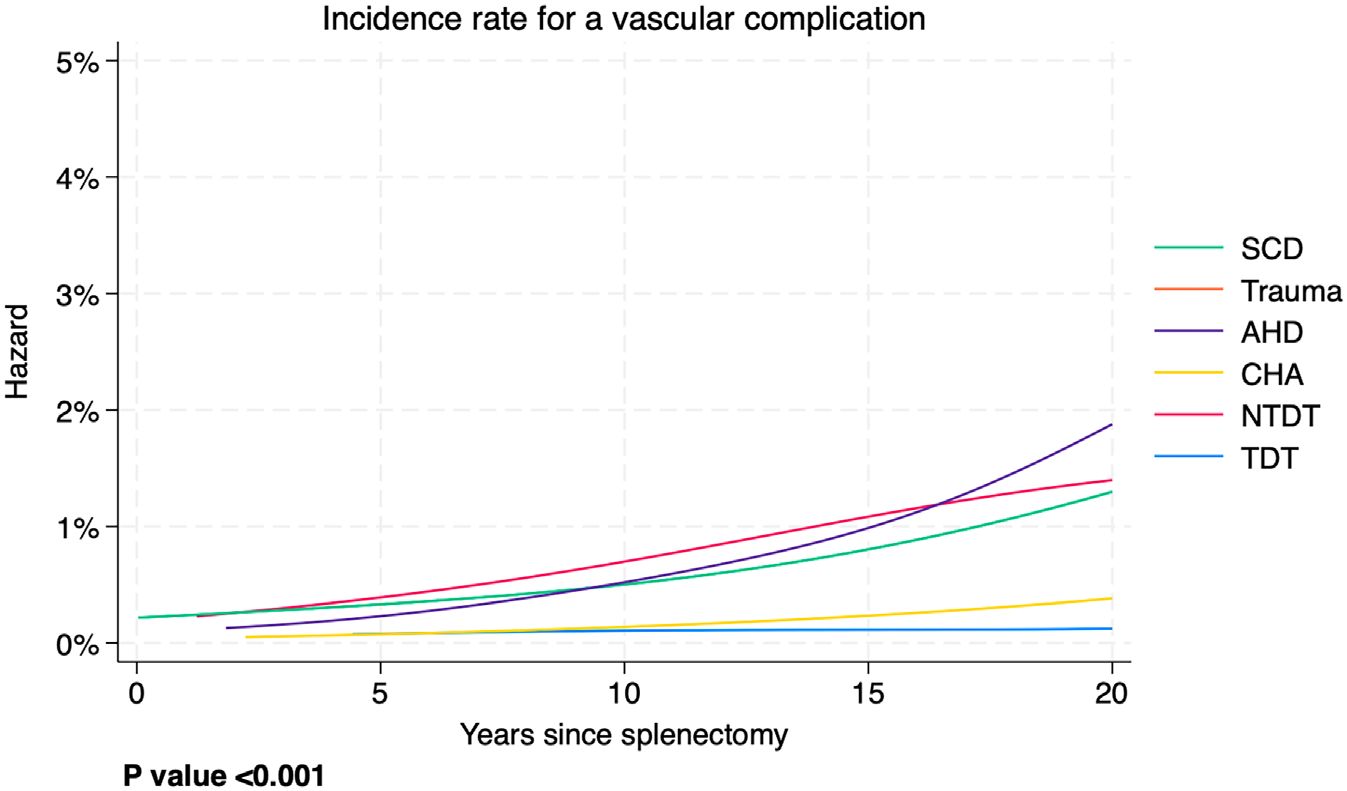
Incidence rate for a vascular complication year by year for each diagnostic group. AHD, autoimmune haematological disease; CHA, congenital haemolytic anaemia; NTDT, non‐transfusion‐dependent thalassaemia; SCA, sickle cell anaemia; TDT, transfusion‐dependent thalassaemia.

Figure 2 shows the risk of a vascular complication year by year for each diagnostic group separately. For example, a non‐transfusion‐dependent thalassaemia (NTDT) patient (red line) who has been free from vascular complications for 15 years after splenectomy, had a risk of about 1% to experience a vascular complication before the 16th year. The most important aspects of this graph are the following: (1) for any disease, the risk of a vascular complication is much lower than the risk of an infectious complication (see Figure 1) and (2) transfusion‐dependent thalassaemia (TDT) and congenital haemolytic anaemia (CHA) patients show very low rates compared to the other patient groups, for whom the risk of vascular complications never decreases over time. The *p* value for a difference between the curves was <0.001, indicating that there is very strong evidence that the risk patterns are different between the disease groups.

**FIGURE 3:**
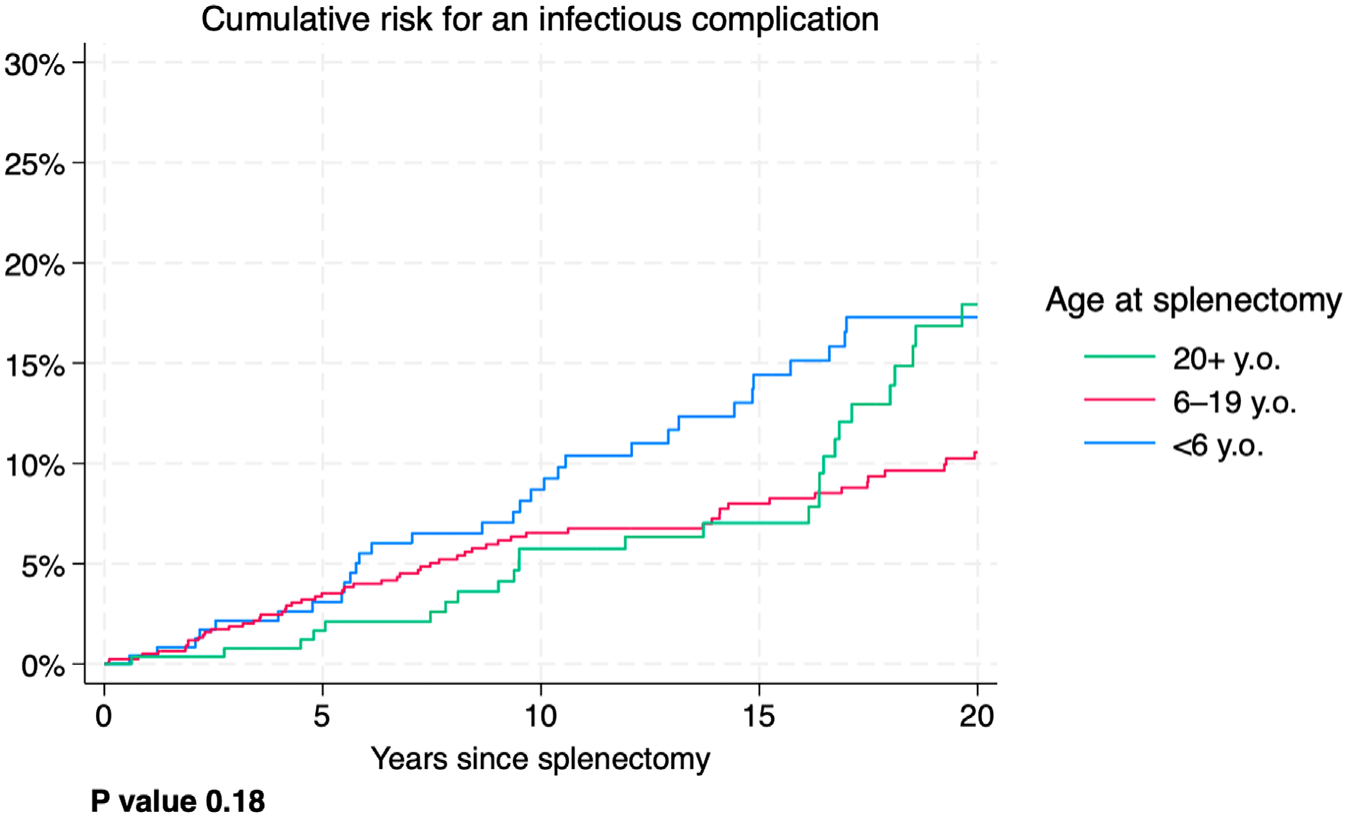
Cumulative risk for an infectious complication after splenectomy by age categories.

Figure 3 shows the risk of an infectious complication within a certain time after splenectomy, by age categories. For example, the probability of an infectious complication within 5 years after splenectomy in subjects splenectomised at age ≥20 (green line) was about 3%, whereas that for the other groups (red and blue lines) was about 4%. The most important aspects of this graph are the following. (1) For all age groups, the risk of developing an infectious complication within the first 2 or 3 years since splenectomy is rather low. (2) Patients in certain age groups seem to have an advantage for certain windows of time. This advantage, however, is cancelled or becomes a disadvantage for later time windows. This shows that the age at splenectomy is not a robust predictor of infectious complications. The *p* value for a difference between the curves was 0.18, indicating that there is no evidence that the risk patterns are different between the age groups.

**FIGURE 4:**
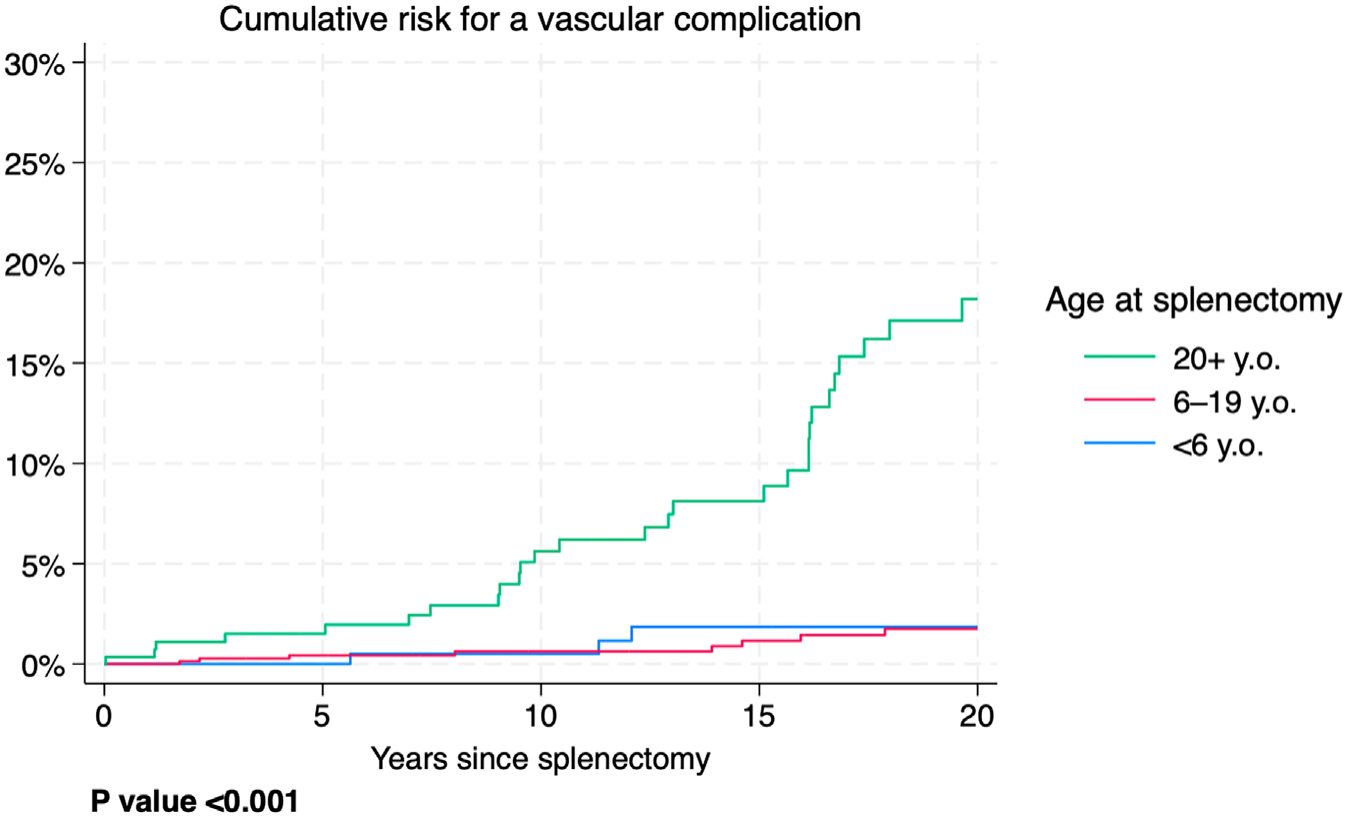
Cumulative risk for a vascular complication after splenectomy by age categories.

Figure 4 shows the risk of a vascular complication within a certain time after splenectomy, by age categories. For example, the probability of a vascular complication within 10 years after splenectomy in patients splenectomised at age ≥20 (green line) was about 5%, whereas that for the other groups (red and blue lines) was close to 0%. The most important aspects of this graph are the following. (1) For all age groups, the risk of developing a vascular complication within the first 2 or 3 years since splenectomy is rather low. (2) Older patients at splenectomy are more likely to experience a vascular complication compared to younger patients. The *p* value for a difference between the curves was <0.001, indicating that there is very strong evidence that the risk patterns are different between the age groups.

Table 2 shows the probability of complications at 10 years following splenectomy for each diagnostic group separately. For infections, the risk ranged from 3.2% (TDT) to 21.4% (SCA). Adjusting for age did not decrease the heterogeneity in those risks. Similarly, for vascular complications, the risk ranged from 0.6% to 3.6% and was not influenced by age at splenectomy. Table 2 therefore shows that the probabilities remain similar after the effect of age was eliminated. Since the crude risks (no adjustment) are very similar to the age‐adjusted risk, the results indicate that age did not influence the overall pattern of risk of complications, and since the risks continued to be very different between the disease groups after the adjustment, our results indicated that the underlying disease is the stronger driver of complications.

**TABLE 2:** Risk of complications and disease‐specific mortality within 10 years after splenectomy.

Disease group	Infectious complication		Vascular complication		Mortality for infectious complication		Mortality for vascular complication	
	Crude	Adjusted for age	Crude	Adjusted for age	Crude	Adjusted for age^a^	Crude	Adjusted for age^a^
TDT	3.2%	3.4%	0.6%	0.6%	1.1%	‐	0.1%	‐
NTDT	4.9%	4.4%	3.4%	2.2%	0.5%	‐	0.2%	‐
SCA	21.4%	22.0%	3.6%	3.5%	0.0%	‐	0.5%	‐
CHA	6.6%	6.8%	1.1%	1.0%	0.0%	‐	0.0%	‐
AHD	10.0%	9.3%	2.8%	2.0%	3.8%	‐	0.0%	‐
Trauma	17.0%	15.6%	3.7%	2.3%	0.0%	‐	0.0%	‐
*p* Value for homogeneity	<0.001	<0.001	<0.001	<0.001	<0.001	‐	<0.001	‐

Abbreviations: AHD, autoimmune haematological disease; CHA, congenital haemolytic anaemia; NTDT, non‐transfusion‐dependent thalassaemia; SCA, sickle cell anaemia; TDT, transfusion‐dependent thalassaemia.

^a^ Adjusted rates could not be estimated due to the scarcity of mortality events.

Table 3 shows the output from a multiple survival model. Three factors were analysed simultaneously: age, sex and underlying disease. The variable age was treated as a continuous variable. Any factor was compared to a baseline category, for example, older age at splenectomy (5‐year increase) versus younger age at splenectomy, males versus females and any diagnostic category versus trauma. Subhazard ratio (SHR) values greater than 1 indicate an increase in risk and values less than 1 indicate a decrease in risk, when the *p* value is statistically significant.

**TABLE 3:** Output from two competing‐risk survival models including interaction terms between age at splenectomy and diagnostic group, adjusted for gender.

Factor and category	Infectious complication				Vascular complication			
	SHR^a^	95% CI		*p*	SHR^a^	95% CI		*p*
Age (5‐year increase)	0.94	0.76	1.16	0.55	**1.82**	**1.45**	**2.28**	**<0.001**
Male versus female	**1.47**	**1.1**	**1.97**	**0.009**	0.77	0.53	1.12	0.18
Diagnostic group								
TDT	**0.07**	**0.01**	**0.35**	**0.001**	2.43	0.42	13.91	0.32
NTDT	**0.15**	**0.03**	**0.76**	**0.022**	**10.54**	**1.84**	**60.49**	**0.008**
SCA	1.25	0.24	6.64	0.79	**8.90**	**1.47**	**53.92**	**0.017**
CHA	0.28	0.06	1.36	0.11	3.72	0.62	22.40	0.15
AHD	0.4	0.07	2.12	0.28	**25.84**	**3.08**	**217.02**	**0.003**
Trauma	1 (baseline category)				1 (baseline category)			
Slope of age in each stratum								
TDT	**1.46**	**1.11**	**1.91**	**0.006**	0.74	0.55	1.00	0.051
NTDT	1.16	0.91	1.48	0.23	**0.68**	**0.53**	**0.88**	**0.003**
SCA	1	0.71	1.4	0.99	0.84	0.65	1.09	0.19
CHA	1.11	0.83	1.47	0.49	0.74	0.57	0.97	0.026
AHD	1.14	0.88	1.47	0.33	**0.53**	**0.35**	**0.80**	**0.003**
Trauma	1 (baseline category)				1 (baseline category)			

Abbreviations: AHD, autoimmune haematological disease; CHA, congenital haemolytic anaemia; CI, confidence interval; NTDT, non‐transfusion‐dependent thalassaemia; SCA, sickle cell anaemia; SHR, subhazard ratio; TDT, transfusion‐dependent thalassaemia.

*Note*: Bold values indicate statistically significant comparisons.

^a^ Mutually adjusted subhazard ratio from competing‐risk survival analysis.

Increasing age at splenectomy was associated with an increased risk of vascular complications (SHR 1.82, *p* < 0.001) but not of infectious complications (SHR 0.94, *p* 0.55).

In males, a higher risk of infections (SHR 1.47; *p* 0.009) but not of vascular complications (SHR 0.77, *p* 0.18) was observed compared to females.

Compared to subjects splenectomised for trauma, a lower risk of infectious complications was observed in TDT (SHR 0.07, *p* 0.001) and NTDT splenectomised patients (SHR 0.15; *p* 0.022), while a higher risk of vascular complications was observed in SCA (SHR 8.9; *p* 0.017), NTDT (SHR 10.54; *p* 0.008) and autoimmune haematological diseases (AHDs) (SHR 25.84; *p* 0.003).

The box ‘Slope of age in each stratum’ indicates the effect of increased age at splenectomy on infectious and vascular complications in each individual disease, compared to trauma. Therefore, increased age at splenectomy increased the risk of infections in TDT (SHR 1.46, *p* 0.006) more than in subjects splenectomised for trauma. Furthermore, in trauma increased age at splenectomy increased the risk of vascular complications more than in AHDs (SHR 0.53, *p* 0.003) and in NTDT (SHR 0.68, *p* 0.003).

This analysis underlines that, after adjustment for diagnosis group and gender, increased age at splenectomy either has no effect or increases the risk of post‐splenectomy complications, but it is never protective. Furthermore, after adjusting for age and gender, each disease has its own risk profile for post‐splenectomy complications, and the risk is differently influenced by age.

## DISCUSSION

Splenectomy can be an urgent life‐saving procedure in certain haematological conditions and in trauma, or it can be a planned operation to alleviate the symptoms of an underlying disease.

The relationship between underlying disease, age at splenectomy, elapsed time since splenectomy and the risk of asplenia‐related complications has always been a point of great interest for researchers and clinicians, as it greatly influences the decision‐making process.

In our study, we investigated all infectious events that required hospitalization, reporting as a final diagnosis meningitis in only 1% of cases, sepsis in 15%, while pneumonia was involved in 42% of cases and other febrile events in 42% of cases. While an admission for infection affected about one in six splenectomy patients in our cohort of splenectomized patients, the low number of meningitis and sepsis compared to total admissions for infections suggests that preventative protocols are effective in curbing the most invasive bacteria.^25^

Our analysis indicated that delaying splenectomy does not reduce the risk of infectious complications in all diseases considered, and the key message is that it is no longer reasonable to postpone splenectomy when clinically indicated. This finding was even more evident in subjects with TDT and partly in NTDT, in which the risk of infectious and vascular complications increased with increasing age at splenectomy, contrary to what is generally and traditionally believed. The immunological impairment and cardiovascular risk increased by long‐term iron overload complications, prolonged oxidative stress and early immune senescence may explain the increasing risk of post‐splenectomy complications in older splenectomised TDT and NTDT patients.^26^, ^27^, ^28^, ^29^ However, in all disorders assessed in this study, the decision to perform splenectomy must depend on the clinical indication and therapeutic goal in the individual disease, regardless of the patient's age. To date, similar results were reported only for sickle cell patients^19^ and for infectious complications.

Our data suggest revising age as a criterion for indication to splenectomy and highlight the importance of weighing the pros and cons of splenectomy in individual diseases, considering the burden and side effects of alternative therapies to splenectomy and the risks associated with spleen‐related complications.

For instance, some forms of congenital haemolytic anaemia, such as severe or moderate hereditary spherocytosis, are maintained on a regular transfusion regimen for several years before splenectomy.^3^ However, in this group of patients, our data showed a high safety profile of splenectomy, a curative procedure that almost completely frees patients from the risk of severe acute anaemia and the need for transfusions.

Treatment protocols for some AHDs, such as immune thrombocytopenia or immune haemolytic anaemia, involve the use of potent immunosuppressive agents for years before splenectomy, administered to delay or avoid the surgical procedure as much as possible.^7^, ^8^, ^29^ The reported infectious risk in patients splenectomised for autoimmune diseases was intermediate, but all reported deaths were associated with infections, with the highest mortality for infections among all disease groups. So, the indication for splenectomy in subjects with AHDs requires particular caution, and in light of these data, the follow‐up and management of infectious events must be particularly aggressive in this group of patients.

In NTDT, the risk of post‐splenectomy complications clearly exceeds the benefit of the increase in the haemoglobin value (generally 1–2 g/dL) obtained by splenectomy, at any age. Chronic anaemia in NTDT patients should be managed with a regular transfusion regimen or new drugs, where available and effective.^30^ However, in some NTDT patients, splenectomy is inevitable when severe hypersplenism and/or symptomatic splenomegaly occur, or anaemia cannot be managed by regular transfusions due to immunization or limited resources, as in low‐income countries, with restricted availability of new expensive drugs, as well. Therefore, patients with NTDT will continue to require splenectomy worldwide. Even in our national database, despite a clear reduction in the number of NTDT patients registered with splenectomy performed from 2010 onwards, compared to previous decades, spleen removal in NTDT continues to be performed even in very recent years. So, it is crucial to consider the specific risk of post‐splenectomy complications in individual diseases in order to implement adequate monitoring and preventive measures.

In subjects with CHA, SCA and trauma, even with very different rates of infection, the lowest in CHA and the highest in SCA, no deaths from infection were reported, according to data published in Italian splenectomized patients with SCA.^32^ These elements must be taken into account in the decision‐making process as splenectomy in these patient groups is often necessary to treat severe complications of the underlying disease.

It should be noted the limited number of patients splenectomised for trauma regularly followed up in specialized centres (51 out 1348 splenectomised subjects). This suggests that patients splenectomised for trauma may not receive updated information on post‐splenectomy risks and may not implement all the preventive measures expected for asplenic patients. To address this critical issue revealed by our data, the collaboration between specialized centres and surgeons or general practitioners is essential to ensure correct management of post‐splenectomy risk in subjects splenectomised for trauma.

The identification of a specific period of increased risk of serious infection after splenectomy is key for clinical practice to appropriately intensify surveillance and prevention. According to our data, in all diseases, the risk of developing an infectious or vascular complication is persistent throughout the observation period and is not limited to the first 2–3 years after splenectomy, as reported in some studies.^31^ Probably, the different methods of recording infectious events in the different studies (analysis of hospital discharge diagnosis versus direct recording of infectious events by the attending physician of the splenectomized patient) explain these differences in the results.

## LIMITATIONS

Our study has some limitations. Our follow‐up period started with splenectomy, and for many patients, we did not have reliable clinical information for the years preceding splenectomy. This can introduce immortal time bias. Such bias is particularly relevant for studies concerning drugs. Immortal time in observational studies can bias the results in favour of the treatment group. To avoid such bias, we should have started the observation time at birth and splitted the records at the time of splenectomy, but as said, for many patients we did not have reliable information for the years before splenectomy and hence we could not deal with immortal time statistically. Nevertheless, our results cannot be explained by immortal time bias. Quite the opposite, immortal time bias reinforces our results. Age was our main exposure variable, and immortal time bias may have artificially increased a protective effect of age. To the contrary, we found that older age at splenectomy was not protective against complications due to splenectomy. Immortal time bias may explain why previous studies found a protective effect of older age at splenectomy.

Our database includes information about prophylactic care, that is vaccines and antibiotics for infections, as well as heparin, anti‐platelets and anti‐coagulants for vascular complications. However, with our observational data, it is impossible to assess the true effect of those drugs for a bias called confounding by indication: doctors may have prescribed these drugs to patients they considered particularly at risk.

The observation time differed for the different conditions, because our data came from a real‐world setting and therefore we could not set an observation time a priori. Furthermore, we could not adjust for some important risk factors for the outcome measures, which could have refined our risk estimates. The limited number of subjects splenectomised for trauma compared to other disease groups reduced the representativeness of this group of asplenic subjects. The post‐splenectomy risk profiling in subjects splenectomised for trauma and for autoimmune disease would be improved with a larger sample size and a longer duration of follow‐up.

Although the number of hospitalizations due to infections and the cause of death was registered for all subjects included in the database, in most cases the pathogen that caused the hospitalization and eventually death due to infection was not reported. This is a typical limitation of real‐life studies, where a responsible pathogen is not identified in approximately 30%–50% of cases of systemic infection.^33^

Another limitation of our study is the absence of a control group. The optimal study design should include a control group of non‐splenectomised patients, with the same underlying disease and with the same baseline characteristics, followed with the same follow‐up compared to splenectomised subjects. However, although ideal, this type of study is unlikely to be feasible and sustainable in the long term.

The strength of our study lies in having systematically observed different disease groups over a long period of time, with data being collected in a uniform and rigorous manner, with stringent outcome definitions, under the control of the physician who directly treated the splenectomised patients. Moreover, our patients were followed up according to protocols that had been previously shared among the different participating centres, thus overcoming the limitation of comparability of the different subcohorts and case histories. Some of our patient records are relatively old. For example, of the 112 recorded deaths, 88 occurred before the records were centralized into our single data centre (year 2014). However, all patient records are of equal quality, as they are derived from official clinical records, and all patients have been prospectively followed since the date of their splenectomy. This is a unique achievement in this field.

In conclusion, we found that the risk of complications and mortality associated with splenectomy in haematological patients is not so much a matter of timing of surgery but rather a matter of underlying disease. The rate and severity of post‐splenectomy complications vary widely among different diseases but are not affected by the age at splenectomy and time elapsed. Reference data can be particularly valuable for comparison with the risk of alternative therapies to splenectomy, in order to reach a clinical decision based on strong and detailed scientific evidence in different haematological diseases.

## AUTHOR CONTRIBUTIONS

M.C. initiated the project, designed the research and wrote the paper with input from other authors. All authors collected data, except A.I.L., and M.C. and A.I.L. analysed the data. All authors approved the paper.

## FUNDING INFORMATION

This work was supported by a grant from the American Society of Hematology (ASH Global Research Award 2019).

## CONFLICT OF INTEREST STATEMENT

All authors declare no conflicts of interest.

## CLINICAL TRIAL REGISTRATION

ClinicalTrials.gov ID: NCT03571399.

## Data Availability

For original data, contact the corresponding author (maddalena.casale@unicampania.it).

## Appendix

*Italian Network of Asplenia*: Piero Farruggia (Palermo), Francesca Fioredda (Genova), Paola Giordano (Bari), Federico Verzegnassi (Trieste), Maria Luisa Casciana (Mantova), Assunta Tornesello (Lecce), Ilaria Capolsini (Perugia), Paolo Grotto (Treviso), Paola Maria Grazia Sanna (Sassari), Luciana Rigoli (Messina), Rosanna Di Concilio (Nocera), Flavia Rivellini (Pagani), Annamaria Pansanisi (Brindisi), Gloria Colarusso (Prato).
